# A Global Burden Perspective on Obstructive Sleep Apnea, Hearing Loss, and Early-Onset Cognitive Decline

**DOI:** 10.3390/neurolint18060117

**Published:** 2026-06-16

**Authors:** Alice Tomaselli, Antonina Luca, Mario Lentini, Jerome Rene Lechien, Federico Mollame, Alberto Caranti, Claudio Vicini, Matteo Lazzeroni, Pasquale Capaccio, Giannicola Iannella, Valentin Favier, Antonino Maniaci

**Affiliations:** 1 Department of Medicine and Surgery, University of Enna Kore, 94100 Enna, Italy; alice.tomaselli@unikorestudent.it (A.T.); antonina.luca@unikore.it (A.L.); mario.lentini@unikore.it (M.L.); federico.mollame@unikorestudent.it (F.M.); 2 Research Study Group of Young-Otolaryngologists, International Federations of Oto-Rhino-Laryngological Societies (YO IFOS), 75001 Paris, France; jerome.lechien@umons.ac.be; 3 Department of Human Anatomy and Experimental Oncology, Faculty of Medicine, UMONS Research Institute for Health Sciences and Technology, University of Mons, 7000 Mons, Belgium; 4 ENT & Oral Surgery Unit, Head and Neck Department, Morgagni-Pierantoni Hospital, AUSL Romagna, 47121 Forlì, Italy; alberto.caranti@gmail.com (A.C.); claudio@claudiovicini.com (C.V.); 5 Department of Otorhinolaryngology & Head and Neck Surgery, Fatebenefratelli Hospital, ASST Fatebenefratelli Sacco, 20121 Milan, Italy; matteo.lazzeroni@unimi.it (M.L.); pasquale.capaccio@unimi.it (P.C.); 6 Department of Biomedical, Surgical and Dental Sciences, University of Milan, 20121 Milan, Italy; 7 Department of Sense Organs, Sapienza University of Rome, 00185 Rome, Italy; giannicola.iannella@uniroma1.it; 8 Otolaryngology—Head and Neck Surgery, Gui de Chauliac Hospital, University Hospital of Montpellier, 34000 Montpellier, France; valentin_favier@hotmail.com

**Keywords:** cognitive decline, obstructive sleep apnea, hearing loss, modifiable risk factors, neurodegeneration

## Abstract

**Background/Objectives**: Cognitive decline and dementia represent a growing global crisis, affecting over 57 million individuals worldwide, projected to exceed 150 million by 2050. The 2024 Lancet Commission identified hearing loss as the single largest modifiable dementia risk factor (~7% population-attributable fraction). Obstructive sleep apnea (OSA), affecting ~936 million adults, is an increasingly recognized contributor yet remains underdiagnosed, especially in low- and middle-income countries (LMICs). This review synthesizes evidence on the global burden of cognitive decline associated with both conditions, evaluates causality debates, and identifies research gaps. **Methods**: Following SANRA guidelines, a search was conducted across PubMed, Scopus, Web of Science, and the Cochrane Library through February 2026. Original studies, systematic reviews, meta-analyses, and WHO/GBD reports were included; editorials and non-English publications were excluded. After deduplication, 3847 records were screened, and 96 studies met the inclusion criteria. **Results**: OSA has been linked to cognitive decline through several plausible mechanisms, including intermittent hypoxia, sleep fragmentation, impaired glymphatic clearance, and amyloid-beta accumulation, though the directionality of these associations requires confirmation from longitudinal studies. Hearing loss contributes to cognitive load, social isolation, and cortical reorganization. Both conditions disproportionately affect LMICs, where access to diagnosis and treatment remains limited. CPAP and hearing rehabilitation show cognitive benefits when initiated early, though evidence for reversing established impairment remains limited. A synergistic interaction between the two conditions is biologically plausible but empirically underexplored. **Conclusions**: OSA and hearing loss are highly prevalent conditions associated with increased dementia risk, though the certainty of causal relationships and the magnitude of intervention effects differ between the two conditions and across the available evidence. Integrated screening and early intervention could yield substantial neuroprotective benefits in high-risk populations and LMICs. Future longitudinal studies should examine combined cognitive trajectories and optimal intervention timing.

## 1. Introduction

Cognitive decline and dementia represent one of the most pressing global health challenges of the twenty-first century. According to the last Global Burden of Disease (GBD) Study, dementia affected approximately 57.4 million individuals worldwide, with projections indicating this number will rise to 152.8 million by 2050 [1]. The economic consequences are staggering, with global costs estimated at 1.3 trillion US dollars in 2019 and expected to surpass 2.8 trillion by 2030 [2]. Beyond financial implications, dementia exerts profound effects on patients, caregivers, and healthcare systems, making the identification of modifiable risk factors a paramount public health priority.

The 2024 Lancet Commission on Dementia Prevention, Intervention, and Care identified fourteen potentially modifiable risk factors that together account for approximately 45% of worldwide dementia cases [3]. Chronic intermittent hypoxia, sleep fragmentation, sympathetic nervous system overactivation, systemic inflammation, oxidative stress, endothelial dysfunction, and impaired glymphatic clearance of neurotoxic metabolites during sleep have been proposed to explain the association between OSA and cognitive decline.

Among these factors, hearing loss emerged as the single largest contributor, responsible for an estimated 8.2% of the population-attributable fraction, followed by low education, cholesterol, visual loss, hypertension, obesity, smoking, depression, physical inactivity, diabetes, social isolation, excessive alcohol consumption, traumatic brain injury, and air pollution [3]. This landmark strongly suggests a proactive approach to dementia prevention targeting reversible determinants across the lifespan.

Obstructive sleep apnea (OSA) has garnered increasing attention as an additional modifiable risk factor for cognitive impairment, though it was not included in the original Lancet Commission framework. Epidemiological data from Benjafield and colleagues estimate that OSA affects approximately 936 million adults aged 30 to 69 years globally, with moderate to severe disease present in 425 million individuals [4]. Despite the high prevalence, OSA frequently remains underdiagnosed, particularly in low- and middle-income countries where access to polysomnography is limited [5]. Several pathophysiological mechanisms have been proposed to explain the association between OSA and cognitive decline, including chronic intermittent hypoxia, sleep fragmentation, sympathetic nervous system overactivation, systemic inflammation, oxidative stress, endothelial dysfunction, and impaired glymphatic clearance of neurotoxic metabolites during sleep [6].

Neuroimaging studies have consistently demonstrated structural brain changes in patients with OSA, including reduced grey matter volume in the hippocampus, frontal cortex, and parietal regions, as well as white matter abnormalities indicative of small vessel disease [7]. A meta-analysis by Leng and colleagues found that individuals with sleep-disordered breathing had a 26% increased risk of cognitive impairment and a similar elevation in dementia risk compared to those without the condition [8]. Furthermore, emerging evidence suggests that OSA may be associated with amyloid-beta and tau accumulation, the pathological hallmarks of Alzheimer’s disease, through disruption of slow-wave sleep and impaired cerebrospinal fluid dynamics [9].

Hearing loss, affecting approximately 1.5 billion people worldwide according to the World Health Organization World Report on Hearing, constitutes the third leading cause of years lived with disability globally [10]. The prevalence increases dramatically with age, affecting more than 65% of adults over 60 years. Longitudinal cohort studies have reported robust associations between hearing impairment and faster cognitive decline. Lin and colleagues reported that older adults with hearing loss experienced cognitive decline 30 to 40% faster than those with normal hearing, with the magnitude of decline proportional to the severity of auditory impairment [11]. The proposed mechanisms underlying this association include increased cognitive load required to process degraded auditory signals, social isolation and withdrawal from cognitively stimulating activities, depression, and cross-modal cortical reorganization with reallocation of neural resources from memory and executive functions toward auditory processing [12]. Despite the substantial independent burdens of OSA and hearing loss, their possible combined effects on cognitive trajectories have received limited systematic investigation, and true synergistic interactions remain unproven. Anatomically and physiologically, these conditions share common risk factors, including ageing, obesity, cardiovascular disease, and diabetes, suggesting possible overlapping pathways toward neurodegeneration [13]. Moreover, both conditions preferentially affect similar demographic groups and are amenable to therapeutic intervention, making their joint examination particularly relevant for preventive neurology strategies.

On this ground, this review aims to synthesize current evidence on the global burden of cognitive decline associated with OSA and hearing loss, critically evaluate debated questions in the literature regarding causality and intervention efficacy, identify knowledge gaps, and propose directions for future research and clinical practice. By adopting a global burden of disease framework, we aim to provide clinicians, researchers, and policymakers with an integrated perspective on two highly prevalent yet frequently overlooked contributors to the dementia epidemic.

## 2. Materials and Methods

To ensure methodological rigour and transparency, we conducted this narrative review in accordance with the Scale for the Assessment of Narrative Review Articles (SANRA) [14]. Specifically, we focused on (i) clearly justifying our aims; (ii) designing a structured and reproducible literature search; (iii) setting explicit inclusion and exclusion criteria; (iv) providing a balanced discussion of conflicting findings; (v) critically evaluating the quality and limitations of the evidence; and (vi) accurately referencing all key statements. While this is not a systematic review or meta-analysis, we structured our approach to maximize transparency and reproducibility. Also, a flow diagram adapted from the PRISMA 2020 format [15] is provided to illustrate the literature search and selection process. A comprehensive literature search was performed across PubMed, Scopus, Web of Science, and the Cochrane Library from inception to February 2026. The literature search was updated before resubmission to capture studies published throughout 2025 and the early months of 2026. Search strings combined both controlled vocabulary terms and free-text keywords encompassing obstructive sleep apnea, sleep-disordered breathing, hearing loss, sensorineural hearing impairment, cognitive decline, dementia, Alzheimer’s disease, disease burden, continuous positive airway pressure, hearing aids, and cochlear implantation.

Search terms included combinations of “obstructive sleep apnea,” “sleep-disordered breathing,” “hearing loss,” “hearing impairment,” “cognitive decline,” “cognitive impairment,” “dementia,” “Alzheimer’s disease,” “global burden of disease,” and “epidemiology.” Reference lists of identified articles were manually screened to capture additional relevant publications. Inclusion criteria encompassed original research articles, systematic reviews, meta-analyses, and authoritative reports from the Global Burden of Disease Study and the World Health Organization (WHO). Editorials, commentaries, and studies not available in English were excluded. Two authors independently screened titles and abstracts, with disagreements resolved through consensus. Given the narrative nature of this review, no formal risk of bias assessment or meta-analytic synthesis was performed. Two authors independently screened titles and abstracts, with disagreements resolved through consensus. Given the narrative review nature, we did not performed formal risk of bias assessment or meta-analytic synthesis. However, we interpreted the evidence according to study design and inference level, distinguishing observational associations, mechanistic plausibility, intervention findings, and unresolved uncertainties. For each research question, the available evidence was appraised by considering study design, sample size, how OSA and hearing loss were measured, the degree of confounder adjustment, consistency of findings across studies, and clinical applicability. Special attention was paid to variability in OSA definitions and severity thresholds, differences in hearing-loss measurement approaches, the diversity of cognitive endpoints used, variation in follow-up duration, and treatment adherence across studies.

To improve readability and to distinguish observational findings, mechanistic evidence, intervention data, and remaining uncertainties, we summarized the main evidence across topics in Table 1.

### Research Questions

The review was structured around the following key debated questions identified in the current literature, with each section addressing controversies, conflicting evidence, and knowledge gaps requiring further investigation. To guide this investigation, the following Research Questions were formulated: A. Is obstructive sleep apnea an independent risk factor for cognitive decline, or are observed associations primarily driven by shared comorbidities? B. Does the severity of obstructive sleep apnea correlate with cognitive impairment and may predict cognitive outcomes? C. Can continuous positive airway pressure therapy prevent or reverse cognitive decline in patients with obstructive sleep apnea? D. Is Hearing Loss a Direct Cause of Dementia, or Do Both Conditions Share Common Underlying Diseases? E. Do hearing aids and cochlear implants reduce the risk of cognitive decline and dementia? F. What are the mechanistic pathways linking hearing loss to neurodegeneration: cognitive load, social isolation, cortical reorganization, or shared neuropathology? G. Is there a synergistic effect between obstructive sleep apnea and hearing loss on cognitive decline? H. What is the true global burden of early-onset cognitive decline attributable to obstructive sleep apnea and hearing loss in low- and middle-income countries? I. What are the optimal timing and target populations for intervention to maximize neuroprotective benefits?

## 3. Results

**Figure 1:**
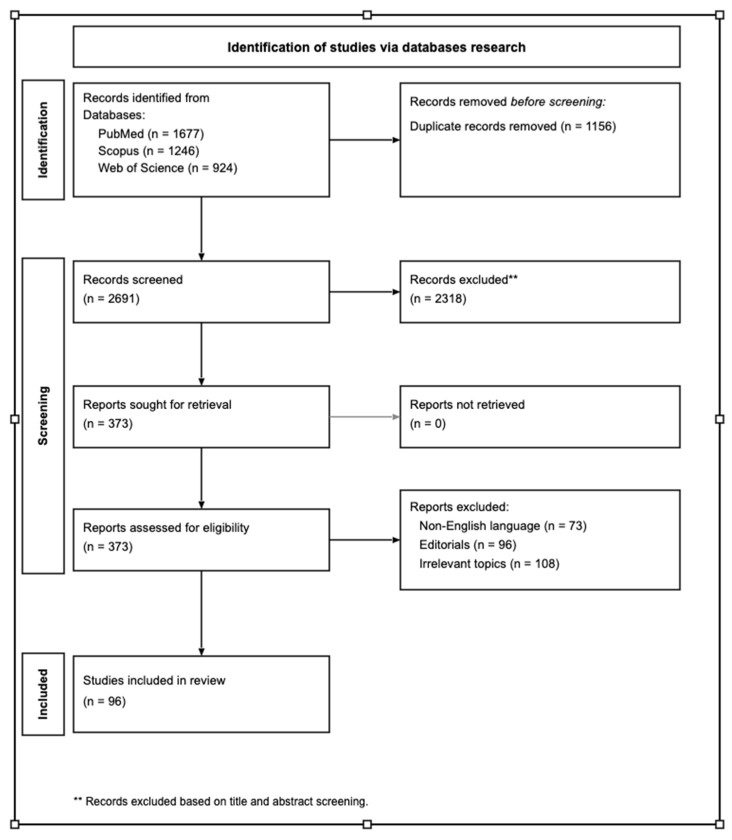
Flow diagram of the literature search and study selection process. Records were identified through database searches of PubMed, Scopus, Web of Science, and the Cochrane Library [15].

The initial database searches yielded a total of 3847 records. After removal of duplicates (*n* = 1156), 2691 titles and abstracts were screened. Following initial screening, 2318 records were excluded as they did not meet the inclusion criteria (non-English language publications, editorials, commentaries, or irrelevant topics). The full texts of 373 articles were assessed for eligibility (Figure 1). Of these, 96 studies met all inclusion criteria and were included in this narrative review, alongside authoritative reports from the Global Burden of Disease Study and World Health Organization guidelines. The identified studies were analyzed and synthesized across nine key debated questions, each addressing a distinct yet interconnected aspect of the relationship between OSA, hearing loss, and cognitive decline. We critically examined the OSA independence as a dementia risk factor, the limitations of current severity metrics, the variable evidence for treatment effects, and the still uncertain interaction between sleep-disordered breathing and hearing impairment. The following sections critically examine the independence of OSA as a dementia risk factor.

### 3.1. Is Obstructive Sleep Apnea an Independent Risk Factor for Cognitive Decline?

The question of whether obstructive sleep apnea represents a truly independent risk factor for cognitive decline or whether the observed associations are driven primarily by shared comorbidities remains one of the most contentious debates in the field. OSA is highly prevalent and frequently undiagnosed, and emerging evidence suggests an association between OSA and depression, cognitive impairment, and dementia [16]. However, the risk of diagnostic misclassification in OSA is bidirectional. Lechat and colleagues demonstrated that single-night polysomnography carries a misclassification probability of approximately 20–50%, particularly in individuals with mild to moderate OSA, raising concerns not only about underdiagnosis but also about overdiagnosis in populations where night-to-night AHI variability is high [17]. Nonetheless, disentangling the unique contribution of OSA from its constellation of associated conditions presents substantial methodological challenges. OSA and dementia share several comorbidities, including diabetes, dyslipidemia, hypertension, and obesity. Notably, it has been proposed that OSA should be included in the metabolic syndrome [18]. Moreover, studies have also linked dementia to specific cardiovascular diseases, such as coronary heart disease, atrial fibrillation, and heart failure. While it is not yet established whether cardiovascular diseases per se increase the risk of dementia or if this is due to shared risk factors, cardiovascular diseases could contribute directly to cognitive decline through cerebral hypoperfusion, hypoxia, embolisms, and infarcts [19].

Recent evidence has emerged challenging the notion that cognitive deficits in OSA patients are merely epiphenomena of comorbid conditions. Patients with OSA often show cognitive deficits, but these have traditionally been attributed to comorbidities. However, recent studies have suggested that OSA may be associated with cognitive decline independently of some measured comorbidities [20].

Interestingly, population-based longitudinal studies have reported that older women with OSA were at greater risk of developing Mild Cognitive Impairment (MCI) or dementia over 5 years than those without OSA (odds ratio 1.85, 95% CI 1.11–3.08) [21]. Recently, results from the longitudinal cohort Alzheimer’s Disease Neuroimaging Initiative (ADNI) reported a significant association of sleep-disordered breathing with MCI and Alzheimer disease. Within the ADNI cohort, OSA was associated with an earlier onset of Alzheimer’s disease in observational analyses, even after adjustment for potential confounding variables including sex, APOε4 genotype, diabetes, depression, body mass index, cardiovascular disease, hypertension, age at baseline, and level of education [21,22].

This direct link is confirmed by a massive systematic review and meta-analysis conducted by Bubu and colleagues, which showed that individuals with sleep apnea face a significantly higher risk of developing neurocognitive disorders (HR 1.43), Alzheimer’s disease (HR 1.28), and Parkinson’s disease (HR 1.54) [23]. These risks are further supported by a recent meta-analysis of 39 cohort studies published by Gong and colleagues, which demonstrated a robust statistical association between all-cause dementia and OSA, reporting a hazard ratio of 1.33 with a 95% confidence interval ranging from 1.09 to 1.61 [24]. Despite several epidemiological studies investigating the link between OSA and cognitive decline, there is still no consensus on whether OSA increases the risk of dementia or not, probably due to methodological issues including differences in OSA definitions, population characteristics (i.e., presence of medical comorbidity), and cognitive outcomes (MCI, dementia, cognitive decline, Alzheimer’s disease), as well as studies design (cross-sectional, cohort) [25].

Although it is still debated whether OSA represents an independent risk factor for cognitive decline, some mechanistic pathways could provide biological plausibility for a causal relationship independent of comorbidities. The key pathophysiologic mechanisms that may underlie this association include hypoperfusion, endothelial dysfunction, and neuroinflammation. Intermittent hypoxia plays a critical role in initiating and amplifying these pathologic processes. Hypoperfusion and impaired cerebral vasomotor reactivity lead to the development or progression of cerebral small vessel disease. Hypoxemia exacerbates these processes, resulting in white matter lesions and neurodegeneration. Moreover, Blood–brain barrier hyperpermeability and neuroinflammation lead to altered synaptic plasticity, neuronal damage, and worsening cerebral small vessel disease [16].

However, OSA is not yet universally recognized as a modifiable risk factor for dementia. Further studies examining at-risk populations (those with subjective cognitive impairment and MCI), as well as accounting for different confounders such as comorbidities, sleep disorders, and medications that might modify or accelerate cognitive decline, are required. In this context, the distinction between OSA with and without significant nocturnal hypoxemia appears particularly relevant, as the hypoxic burden (defined as the degree and duration of nocturnal oxygen desaturation) may represent a more precise predictor of cognitive outcomes than the AHI alone [26]. Future prospective studies on individuals with normal cognition, no other medical comorbidities, polysomnographic assessment of OSA, and longitudinal cognitive outcomes evaluation are needed to establish whether OSA constitutes an independent, modifiable risk factor of cognitive decline.

### 3.2. Does the Severity of Obstructive Sleep Apnea Correlate with Cognitive Impairment, and May Predict Cognitive Outcomes?

The relationship between OSA severity and cognitive impairment has been the subject of extensive investigation, yet a clear linear correlation has not been consistently established. The apnea–hypopnea index (AHI), defined as the sum of apneas and hypopneas per hour, is currently the most commonly used clinical indicator to assess the severity of OSA. However, it only reflects the frequency of respiratory events and ignores the severity of respiratory-related hypoxic events [26]. This fundamental limitation has prompted researchers to explore alternative metrics that may better capture the pathophysiological burden of OSA on neurocognitive function.

Studies have demonstrated that the percentage of cases showing abnormal cognitive scores increases with OSA severity from moderate (AHI 15–30 events/h) to severe (AHI 30–50 events/h) to very severe (AHI > 50 events/h) OSA (25%, 85.7%, and 100%, respectively) [27]. Moreover, there was a significant negative correlation between OSA severity (AHI) and the Montreal Cognitive Assessment (MoCA) score [28]. Similarly, when the AHI was used to categorize the severity of sleep apnea, the prevalence rate of MCI was highest in people with severe OSA (50.4%) and lowest in people with mild OSA (11.2%) [29]. MCI was present in 47.9% of a large sleep-clinic cohort, increasing to greater than 55.3% in patients with moderate and severe OSA. Moderate and severe OSA were independently associated with greater than 70% higher odds for MCI compared with patients with no OSA [30].

**Figure 2:**
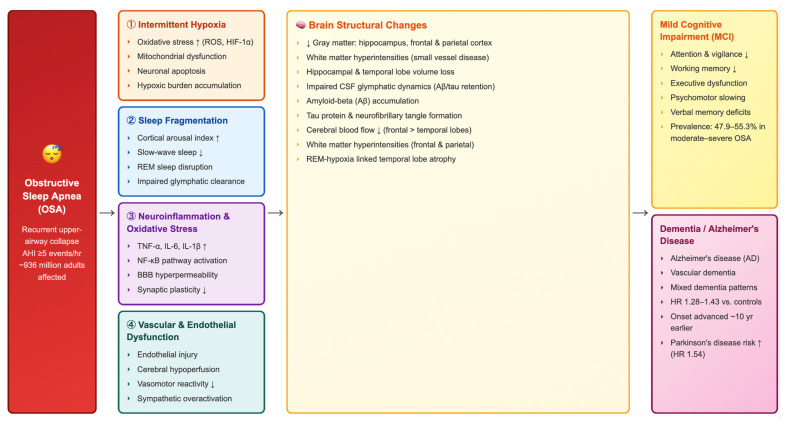
Mechanistic pathways linking obstructive sleep apnea (OSA) to cognitive decline and dementia. Legend: The figure illustrates four distinct mechanistic pathways through which OSA contributes to the development of mild cognitive impairment (MCI) and dementia, including Alzheimer’s disease (AD). Pathway ① depicts intermittent hypoxia-induced oxidative stress and neuronal injury. Pathway ② represents sleep fragmentation with subsequent disruption of sleep architecture and glymphatic clearance failure. Pathway ③ encompasses neuroinflammation and oxidative stress, leading to blood–brain barrier (BBB) disruption and synaptic dysfunction. Pathway ④ illustrates vascular and endothelial dysfunction resulting in cerebral hypoperfusion and small vessel disease. All four pathways converge on brain structural changes as an intermediate endpoint, ultimately progressing toward MCI and, subsequently, dementia/AD. Solid arrows indicate direct causal or mechanistic relationships between stages. Abbreviations: OSA stands for Obstructive Sleep Apnea. AHI stands for Apnea-Hypopnea Index. ROS stands for Reactive Oxygen Species. HIF-1α stands for Hypoxia-Inducible Factor 1-alpha. REM stands for Rapid Eye Movement sleep. CSF stands for Cerebrospinal Fluid. Aβ stands for Amyloid-beta protein. TNF-α stands for Tumor Necrosis Factor-alpha. IL-6 stands for Interleukin-6. IL-1β stands for Interleukin-1 beta. NF-κB stands for Nuclear Factor kappa B. BBB stands for Blood-Brain Barrier. MCI stands for Mild Cognitive Impairment. AD stands for Alzheimer’s Disease. HR stands for Hazard Ratio, and yr stands for year.

Despite these associations, any severity grading from mild to severe usually shows linear correlation with physiological consequences, morbidity or mortality. However, it is not true for OSA severity grading based on AHI score [27]. Patients with the same AHI values can experience varying levels of hypoxemia and sleep fragmentation, leading to inconsistent results when correlating AHI severity with multiple cognitive endpoints [31]. This heterogeneity suggests that the AHI alone may be insufficient for predicting neurocognitive outcomes (Figure 2). Part of this inconsistency may stem from the inherent limitations of the AHI as a metric. It does not capture event duration, the degree of oxygen desaturation reached during each episode, the sleep stages in which events occur, the intensity of cortical arousal, or the total cumulative hypoxic load over time. As a result, two patients sharing an identical AHI may be exposed to quite different neurobiological insults, which likely contributes to the variability seen in cognitive outcomes across studies.

Hypoxemia is a key factor in the development of cognitive impairment in patients with OSA, which can impair cognitive function through a variety of mechanisms, including oxidative stress, inflammation, cerebral hypoperfusion, metabolic dysfunction, and sympathetic activation [26]. Emerging evidence points to hypoxemia-related parameters as potentially superior predictors of cognitive outcomes. Previous studies associated the elevated oxygen desaturation index (≥15 events/h) and the high percentage (>7%) of sleep time in apnea or hypopnea with MCI or dementia (OR = 1.71, 95% CI 1.04–2.83) and dementia (OR = 2.04, 95% CI 1.10–3.78, respectively) [32]. Measures of sleep fragmentation (e.g., arousal index and wake after sleep onset) or sleep duration (e.g., total sleep time) were not reported to be associated with cognitive impairment.

Other variables associated with cognitive decline were measures of arousal index (cut-off: ≥28 events/h, OR: 5.67), sleep mean SpO2 (cut-off: ≤92%, OR: 3.52), 3% oxygen desaturation index (cut-off: ≥27 events/h, OR: 3.75), and sleep time spent under 90% SpO2 (cut-off: ≥9%, OR: 3.16) [31].

The hypoxic burden, a recently developed metric, represents a promising advancement in OSA characterization. The hypoxia burden is a new measurement that has been derived from the results of sleep apnea monitoring in recent years. Compared to the AHI, it better integrates the pathological mechanisms characterizing OSA. It is therefore a promising tool for the identification of people at high risk of OSA and is useful in the prediction of risk of cardiovascular events [26]. However, no relationship was found between indicators such as sleep parameters, AHI, T90, T88, ODI, min SaO_2_, mean SaO_2_ and cognitive impairment after adjusting a series of covariates. Compared with traditional PSG hypoxic parameters, the hypoxic burden may reflect the severity of OSA-specific hypoxemia more comprehensively [26].

The timing and sleep stage specificity of hypoxemia appear particularly relevant. Indeed, a link between low oxygen levels during the rapid-eye-movement (REM) stage of sleep and white matter hyperintensities and temporal lobe atrophy in older adults has been reported [33]. Moreover, it seems that lower oxygen levels during REM sleep, not just the number of breathing interruptions, were most strongly linked to increased white matter hyperintensities, especially in the frontal and parietal lobes, highly active brain lobes during REM sleep and especially vulnerable to low oxygen [33]. Furthermore, previous studies reported that apnea events during REM contribute to verbal memory decline, especially among individuals with an increased familial risk of Alzheimer’s disease [34].

Sleep fragmentation due to frequent arousal represents another pathophysiological pathway linking OSA to cognitive decline [35]. Recently, a novel EEG-derived metric that quantifies frequency-specific power shifts during arousals has been proposed as a possible biomarker of cognitive impairment related to OSA. Arousal intensity increases with greater oxygen desaturation and deeper sleep stages, reflecting graded cortical responses to respiratory events [36].

### 3.3. Can Continuous Positive Airway Pressure Therapy Prevent or Reverse Cognitive Decline in Patients with Obstructive Sleep Apnea?

Continuous positive airway pressure (CPAP) remains the gold-standard treatment for moderate-to-severe OSA, effectively eliminating airway obstruction, normalizing oxygen saturation, and reducing sleep fragmentation [37,38]. However, whether CPAP therapy can reverse established cognitive deficits or prevent further decline remains debated. Indeed, outcomes vary based on treatment duration, adherence levels, cognitive domains assessed, and patient characteristics. Several studies have demonstrated cognitive improvements following CPAP therapy. CPAP treatment in OSA has shown improvement in various neuropsychological tests, including attention, psychomotor speed, executive functioning, and verbal and visual memory. In this clinical context, patient compliance emerges as a decisive factor; accumulating observational data indicate that sustained adherence to CPAP therapy yields a significantly lower incidence of long-term cognitive impairment and dementia when contrasted directly with non-adherent patient cohorts. These cognitive improvements can be seen within weeks of CPAP therapy, although the most prominent effects are usually seen after 3 months of treatment [37]. Among patients with OSA, 3 months’ treatment with CPAP compared with sham CPAP resulted in significant improvement in a composite measure of neurocognitive function, including attention, psychomotor speed, executive functioning, and nonverbal delayed recall, and a few additional neurocognitive measures [38].

The MERGE trial, a large multicenter randomized controlled study, examined whether CPAP therapy would mitigate cognitive decline over 12 months in patients with comorbid OSA and MCI. However, the results were less encouraging. A 12-month CPAP treatment did not result in significant cognitive changes in primary (i.e., memory) or secondary cognitive outcome measures [39]. However, it should be noted that the mean time of CPAP use during the MERGE trial was 2.9 h per night, which may have been insufficient to produce cognitive benefits. Studies with a mean night of CPAP use of 3.5 h per night, leaving nearly half of the night’s sleep untreated, were associated with poor cognitive outcomes [40,41]. These findings underscore that inadequate CPAP use may account for negative results in some intervention studies [42].

The timing of intervention appears to influence treatment efficacy. Early treatment initiation may be more beneficial than delayed intervention after cognitive impairment has become established. OSA treatment with CPAP in the early stages of the disease could be critical in reducing the risk of cognitive decline, suggesting that preventive approaches may be more effective than attempting to reverse existing deficits [43,44].

Neuroimaging studies supported the beneficial role of early OSA treatment in improving cognitive outcomes. In particular, 12 months of CPAP treatment significantly increased hippocampal volume and improved memory performance in OSA patients, suggesting neuroplastic recovery with sustained treatment [45]. White matter integrity, as measured by diffusion tensor imaging, has also been shown to improve following CPAP therapy, particularly in regions affected by chronic intermittent hypoxia [46].

The cognitive domains responsive to CPAP treatment vary across studies. A meta-analysis revealed that CPAP therapy produced significant improvements in attention and vigilance, with moderate effect sizes. However, improvements in memory and executive function were less consistent, with some studies showing benefits while others reported no significant changes [47]. The heterogeneity in findings may reflect differences in baseline cognitive status, OSA severity, treatment duration, and methodological variations across studies.

Long-term observational studies have examined whether CPAP treatment reduces dementia risk. In a large retrospective cohort, patients with OSA who were adherent to CPAP therapy had a significantly lower risk of developing dementia compared to non-adherent patients, with a hazard ratio (HR) of 0.78 after adjusting for confounding variables [48]. Another population-based study found that untreated OSA was associated with earlier onset of MCI by approximately 10 years compared to individuals without sleep-disordered breathing, and that CPAP use was associated with delayed MCI onset [21].

Despite these promising findings, systematic reviews have highlighted the limitations of current evidence. Many randomized controlled trials have been underpowered, of short duration, or compromised by poor CPAP adherence. While there are many short-term studies of CPAP treatment in OSA patients that have found some cognitive improvements with CPAP use, predominantly in attention and vigilance, few long-term follow-up studies of CPAP use in OSA have been performed [49]. Future research should focus on identifying patient subgroups most likely to benefit from treatment and developing strategies to optimize adherence.

The absence of clear benefit in some trials is unlikely to reflect a true null effect and may instead be explained by several methodological factors. Poor adherence is a consistent problem when patients use CPAP for fewer than four hours per night, a substantial portion of sleep-related hypoxemia remains untreated. Beyond adherence, many trials enrolled patients with advanced or already established cognitive impairment, a stage at which underlying neurodegeneration may be too far progressed for symptomatic recovery. Short follow up windows may also miss delayed neuroprotective effects, and the heterogeneity of cognitive endpoints across trials makes cross study comparisons difficult.

CPAP therapy demonstrates potential to improve certain cognitive functions in OSA patients, particularly attention and vigilance, especially when treatment is initiated early and used consistently. However, evidence for reversing established cognitive impairment remains limited, emphasizing the importance of early diagnosis and treatment to prevent irreversible neurocognitive consequences.

### 3.4. Is Hearing Loss a Direct Cause of Dementia, or Do Both Conditions Share Common Underlying Diseases?

The relationship between hearing loss and cognitive impairment is complex, and recent evidence suggests that OSA may serve as a common underlying factor linking these two conditions through shared pathophysiological mechanisms.

A prospective study following older adults over 12 years found that individuals with hearing loss had a 24% increased risk of incident cognitive impairment compared to those with normal hearing, with the risk increasing proportionally to the severity of hearing loss [11]. Furthermore, hearing impairment was associated with accelerated cognitive decline, with hearing impaired individuals experiencing a 30–40% faster rate of cognitive decline compared to their normal-hearing counterparts [50]. These findings have been replicated across diverse populations and study designs, establishing hearing loss as a robust predictor of cognitive outcomes.

Several mechanisms have been proposed to explain the hearing cognition relationship. The cognitive load hypothesis suggests that degraded auditory input requires increased cognitive resources for speech perception, diverting capacity from other cognitive processes such as memory encoding. This constant effortful listening may lead to structural brain changes over time, particularly in regions involved in auditory and cognitive processing [51]. The cascade hypothesis proposes that hearing loss leads to social isolation and reduced environmental stimulation, which in turn accelerates cognitive decline through decreased neural activity and engagement [52]. Additionally, a common cause hypothesis suggests that both hearing loss and cognitive decline may share underlying pathological processes, such as vascular dysfunction, neurodegeneration, or chronic inflammation.

Emerging evidence indicates that OSA may represent a critical link between hearing loss and cognitive impairment. OSA is increasingly recognized as an independent risk factor for sensorineural hearing loss, with multiple studies demonstrating higher prevalence of hearing impairment among OSA patients, with OSA patients had significantly higher risk of hearing loss compared to controls [53]. The proposed mechanisms include cochlear hypoxia due to intermittent oxygen desaturation, microvascular dysfunction affecting the delicate blood supply to the inner ear, and systemic inflammation damaging the auditory pathway [54].

The cochlea is particularly vulnerable to hypoxic injury due to its high metabolic demands and limited vascular supply. The stria vascularis, responsible for maintaining the endocochlear potential essential for auditory transduction, requires continuous oxygen delivery to function properly. Chronic intermittent hypoxia characteristic of OSA can disrupt this delicate system, leading to hair cell damage and progressive hearing loss [55]. Studies have demonstrated that OSA severity, particularly measures of nocturnal hypoxemia such as oxygen desaturation index and time spent below 90% saturation, correlates with the degree of hearing impairment [56].

Neuroimaging studies have revealed overlapping patterns of brain changes in both hearing loss and OSA. Both conditions are associated with reduced grey matter volume in temporal lobe structures, including the hippocampus and auditory cortex. Furthermore, white matter hyperintensities, indicative of small vessel disease, are increased in patients with hearing loss and OSA alike [57]. These shared neuroanatomical features suggest common vascular or inflammatory pathways contributing to both auditory and cognitive dysfunction.

The relationship between OSA, hearing loss, and cognition may be bidirectional and synergistic. Hearing loss can exacerbate the cognitive burden of OSA by impairing communication and social engagement, while OSA-related sleep fragmentation may further compromise auditory processing during sleep, a period critical for neural plasticity and memory consolidation [58]. A study examining the combined effects of OSA and hearing loss on cognitive function found that individuals with both conditions had significantly worse cognitive performance than those with either condition alone, suggesting an additive or synergistic effect [59].

Treatment implications of this triadic relationship are significant. CPAP therapy for OSA has been shown to improve hearing thresholds in some patients, particularly those with more severe nocturnal hypoxemia, suggesting that addressing OSA may provide auditory benefits [60]. Conversely, hearing aid use has been associated with reduced rates of cognitive decline in hearing-impaired individuals, potentially by reducing cognitive load and improving social engagement. The Ageing and Cognitive Health Evaluation in Elders (ACHIEVE) trial, a randomized controlled study, demonstrated that hearing intervention significantly reduced cognitive decline over three years in at-risk older adults [61].

The clinical implications of these interconnected relationships are profound. Comprehensive assessment of older adults presenting with cognitive complaints should include evaluation for both OSA and hearing loss, as addressing these potentially modifiable factors may offer opportunities for intervention. Screening for hearing impairment in sleep clinic populations and, conversely, screening for sleep-disordered breathing in audiology settings may help identify individuals at elevated risk for cognitive decline [62]. Recognizing and treating both conditions may provide synergistic benefits for preserving cognitive function in ageing populations.

### 3.5. Do Hearing Aids and Cochlear Implants Reduce the Risk of Cognitive Decline and Dementia?

The potential for hearing rehabilitation to mitigate cognitive decline has garnered substantial research attention, driven by the recognition that hearing loss represents a modifiable risk factor for dementia. Hearing aids and cochlear implants restore auditory input, potentially reversing the cascade of events linking hearing loss to cognitive impairment. However, the evidence regarding their efficacy in preserving cognitive function is growing.

Observational studies have consistently suggested a protective effect of hearing aid use on cognitive outcomes. A large population-based study examining over 400,000 participants found that hearing aid use was associated with a 19% reduction in the risk of long-term cognitive decline compared to non-users with hearing loss [63]. Similarly, an analysis of the UK Biobank data demonstrated that hearing aid users had cognitive scores comparable to individuals without hearing impairment, while non-users with hearing loss showed significantly worse performance across multiple cognitive domains [64]. These findings suggested that hearing aids might effectively neutralize the excess dementia risk associated with hearing loss.

The mechanisms by which hearing aids may protect cognition are multifaceted. By amplifying sound and improving speech intelligibility, hearing aids reduce the cognitive load required for auditory processing, freeing cognitive resources for other mental operations such as working memory and executive function [65]. Furthermore, improved hearing facilitates social engagement and communication, counteracting the social isolation and reduced environmental stimulation that may accelerate cognitive decline. Hearing aids may also promote neuroplasticity by restoring auditory input to deprived neural pathways, potentially reversing maladaptive brain reorganization associated with hearing loss [66].

The ACHIEVE trial represents the most rigorous examination of hearing intervention effects on cognitive decline to date. This multicenter randomized controlled trial enrolled 977 older adults aged 70–84 years with untreated hearing loss and randomized them to either a hearing intervention including hearing aids and audiologic counselling, or a health education control group. Crucially, within the cohort of participants recruited from the Atherosclerosis Risk in Communities (ARIC) study who presented with a higher baseline vulnerability to cognitive impairment, the audiologic intervention achieved a striking 48% reduction in cognitive decline over a three-year follow-up period compared to the health education control group [61]. This landmark finding provided the first high-quality randomized evidence that treating hearing loss can slow cognitive decline in at-risk populations.

However, the overall ACHIEVE trial results were more nuanced. Indeed, results pertaining to the whole ACHIEVE cohort, which included both ARIC participants and de novo recruits, the difference in cognitive decline between groups was not statistically significant. Thus, according to the ACHIEVE study, the hearing intervention significantly reduced 3-year cognitive change among older adults at increased risk for cognitive decline but not in healthy adults with low dementia risk [67]. These findings suggest that hearing rehabilitation may be particularly beneficial for individuals already experiencing cognitive vulnerability, rather than providing universal protection against age-related cognitive decline.

Cochlear implants, which provide direct electrical stimulation to the auditory nerve, represent the standard of care for individuals with severe-to-profound hearing loss who do not benefit adequately from hearing aids. Studies examining cognitive outcomes following cochlear implantation have generally shown positive results. A systematic review and meta-analysis found that cochlear implant recipients demonstrated significant improvements in global cognitive function, attention, and executive function at 12 months post-implantation [68]. The magnitude of improvement was often greater than that observed with hearing aids, possibly reflecting the more complete restoration of auditory input achieved with cochlear implants.

Long-term follow-up studies of cochlear implant recipients have provided encouraging data on dementia prevention. A retrospective cohort study found that cochlear implant users had a significantly lower incidence of dementia compared to matched controls with severe hearing loss who did not receive implants (adjusted HR 0.79) [69]. Another investigation demonstrated that cognitive trajectories stabilized or improved following cochlear implantation, with the greatest benefits observed in patients who had experienced the most rapid pre-operative cognitive decline [70].

The timing of intervention appears critical for maximizing cognitive benefits. Studies suggest that earlier intervention, before hearing loss has led to significant auditory deprivation and associated brain changes, may yield superior outcomes. Adults who received hearing aids within the first few years after developing hearing loss showed better-preserved cognitive function compared to those with prolonged, untreated hearing loss [71]. Similarly, shorter duration of deafness before cochlear implantation has been associated with better post-operative cognitive outcomes, emphasizing the importance of timely intervention [72].

Patient adherence and consistent device use are essential for realizing cognitive benefits. Studies reported that hearing aids must be worn consistently, typically more than 4–6 h daily, to produce meaningful cognitive protection. Inconsistent use diminishes the potential benefits, as intermittent auditory deprivation continues to exert negative effects on neural plasticity and cognitive reserve [73]. Strategies to improve adherence, including proper fitting, counselling, and follow-up, are therefore necessary for cognitive preservation.

Interestingly, specific cognitive domains show differential responsiveness to hearing intervention. Improvements in processing speed and attention are most consistently observed following hearing aid fitting or cochlear implantation, likely reflecting reduced listening effort and freed cognitive resources. Benefits for memory and executive function are more variable, with some evidence showing significant improvements while others report modest or no changes [74]. The heterogeneity in findings may reflect differences in intervention timing, baseline cognitive status, and the sensitivity of cognitive assessments employed.

However, accumulating evidence supports the cognitive benefits of hearing rehabilitation through hearing aids and cochlear implants. Therefore, timely and consistent use of hearing devices could represent a promising strategy for preserving cognitive function in individuals with hearing loss, particularly those with elevated baseline dementia risk.

### 3.6. What Are the Mechanistic Pathways Linking Hearing Loss to Neurodegeneration: Cognitive Load, Social Isolation, or Cortical Reorganization?

**Figure 3:**
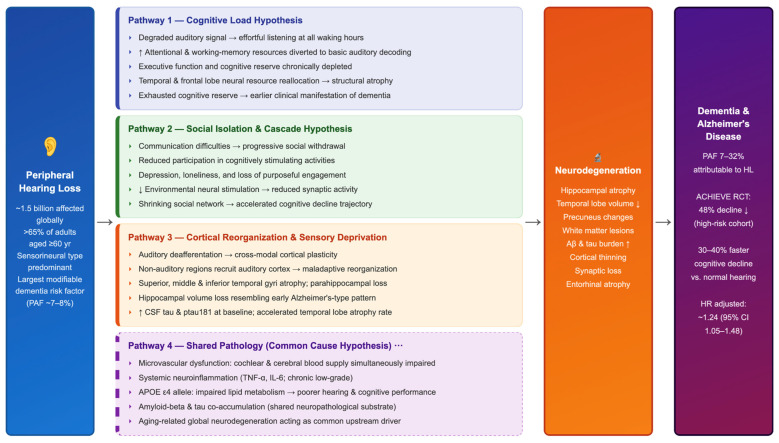
Mechanistic pathways linking peripheral hearing loss to cognitive decline and dementia. Legend: The figure illustrates four mechanistic pathways through which peripheral hearing loss may contribute to neurodegeneration and dementia. Pathway 1 (Cognitive Load) depicts how the chronic compensatory cognitive effort required for auditory processing depletes cognitive resources, accelerating cognitive decline. Pathway 2 (Social Isolation Cascade) represents the behavioural withdrawal associated with hearing loss, leading to social isolation, depression, and reduced cognitive stimulation. Pathway 3 (Cortical Reorganization) illustrates how auditory deafferentation induces maladaptive cortical reorganization and temporal lobe atrophy. Pathway 4 (Shared Pathology) is represented by a dashed border to indicate that microvascular dysfunction, neuroinflammation, genetic predisposition (APOE ε4), and ageing-related neurodegeneration may independently and simultaneously underlie both hearing loss and cognitive decline, without a strict directional causal relationship between the two conditions. All pathways converge on neurodegeneration as a shared intermediate endpoint, ultimately leading to dementia/AD. Solid arrows indicate direct mechanistic or causal progression; the dashed border in Pathway 4 denotes a parallel or bidirectional relationship rather than a linear causal pathway. Abbreviations: PAF stands for Population Attributable Fraction. HL stands for Hearing Loss. RCT stands for Randomized Controlled Trial. Aβ stands for Amyloid-beta protein. HR stands for Hazard Ratio. CI stands for Confidence Interval. CSF stands for Cerebrospinal Fluid. TNF-α stands for Tumor Necrosis Factor-alpha. IL-6 stands for Interleukin-6. APOE ε4 stands for Apolipoprotein E epsilon 4 allele. yr stands for year.

The hypothesized mechanistic pathways through which hearing loss could contribute to increased dementia risk include the effects of hearing on greater cognitive load, changes in brain structure and function, and decreased social engagement. Understanding these mechanisms is essential for developing targeted interventions to mitigate the cognitive consequences of hearing impairment (Figure 3).

#### 3.6.1. Direct Mechanistic Pathways

The cognitive load hypothesis represents one of the most widely discussed mechanistic pathways. The cognitive load theory proposes that hearing loss probably increases the cognitive effort required to process and understand speech, since reduced or distorted sensory input will require the brain to work harder. When auditory signals are degraded, individuals must allocate additional cognitive resources—particularly attention, working memory, and executive function—toward the basic task of auditory perception. With hearing loss, this increased cognitive and listening effort may increase cognitive load at any time of day, as the auditory system never “turns off,” thereby drawing on the cognitive buffer of the individual and their ability to compensate for cognitive changes and resulting in earlier presentation of clinical symptoms and dementia. This constant diversion of cognitive resources may eventually deplete cognitive reserve, leaving fewer resources available for higher-order cognitive processes and accelerating the trajectory toward clinical dementia [12]. The sensory deprivation hypothesis proposes that reduced auditory input leads to structural and functional changes in the brain. Hearing loss can lead to deafferentation-induced atrophy of frontotemporal brain regions and dysregulation of cognitive control networks from increased listening effort. Neuroimaging studies supported this mechanism. Individuals with hearing impairment, compared to those with normal hearing, had accelerated total volume reduction as well as superior, middle, and inferior temporal gyri and parahippocampus atrophy, very close to those observed in Alzheimer’s disease [75]. The cascade hypothesis extends the sensory deprivation model by emphasizing the downstream social and psychological consequences of hearing loss. Social isolation and loneliness are hypothesized potential mechanisms through which hearing loss may be associated with worsened cognitive and mental health. Individuals with hearing impairment often experience communication difficulties that lead to withdrawal from social situations, reduced engagement in cognitively stimulating activities, and increased feelings of loneliness and depression. Interventions to improve the social networks of older adults with hearing impairment are likely to be beneficial in preventing cognitive decline. This pathway is particularly important given that social engagement and cognitive stimulation are known protective factors against dementia [76].

#### 3.6.2. Shared Pathology Mechanisms

Alternative explanations suggest that hearing loss and cognitive decline may share underlying pathophysiological processes rather than having a direct causal relationship. Vascular dysfunction represents one plausible common cause, as both the cochlea and the brain are highly dependent on adequate blood supply and are vulnerable to microvascular damage. The APOE ε4 allele, the strongest genetic risk factor for Alzheimer’s disease, has also been associated with poorer hearing performance and increased hearing difficulty, suggesting shared biological pathways such as lipid metabolism, neuroinflammation, or vascular mechanisms. This genetic overlap suggests that some individuals may be predisposed to both auditory and cognitive dysfunction [77].

#### 3.6.3. Evidence for Multiple Coexisting Mechanisms

Recent research suggests that both direct causal pathways and shared pathology mechanisms may operate simultaneously, contributing to the complexity of the hearing–dementia relationship. Emerging evidence links hearing loss to Alzheimer’s disease pathology, specifically the accumulation of tau protein and amyloid-beta plaques. Age-related hearing loss was associated with higher CSF (Cerebrospinal Fluid) levels of tau or ptau181 at baseline, as well as faster elevation rates of these two types of biomarkers. Although the baseline volume of the hippocampus and entorhinal cortex was higher in individuals with hearing loss, these two regions displayed significantly accelerated atrophy in individuals with hearing loss. These findings suggest that hearing loss may accelerate the neurodegenerative processes characteristic of Alzheimer’s disease, although the directionality of this relationship remains under investigation [78].

Moreover, degraded auditory input places increased demands on hippocampal processing for speech in noise comprehension, potentially accelerating pathological changes in this vulnerable region [79].

Recent neuroimaging research has identified specific patterns of brain changes associated with hearing loss. Poor hearing performance was significantly associated with lower volume of the temporal cortex, including the superior temporal auditory association cortical areas, hippocampus and precuneus, which are the most vulnerable brain regions related to dementia. Mediation analyses have demonstrated that these structural brain changes partially explain the relationship between hearing impairment and cognitive decline, supporting the role of neurodegeneration in linking auditory and cognitive function [80].

However, not all studies support a direct causal link between hearing loss and Alzheimer’s pathology. The heterogeneity in findings suggests that multiple mechanisms may operate in different individuals or populations, and that the hearing–dementia relationship may not be exclusively mediated through traditional Alzheimer’s pathology [81].

#### 3.6.4. Integrated Understanding

The mechanisms linking hearing loss to cognitive decline are likely not mutually exclusive. Multiple pathways, such as shared neuropathology, cognitive load, and increased loneliness, could likely co-exist and synergistically contribute to accelerated cognitive decline in individuals with hearing loss. Individual variability in genetic susceptibility, vascular health, cognitive reserve, and social circumstances may determine which pathways predominate in any given person, explaining the heterogeneity observed in clinical and research settings [82]. Overall, the hearing loss–cognition relationship is best interpreted as multifactorial, involving cognitive load, sensory deprivation, social isolation, and shared biological vulnerability rather than a single linear causal pathway. Understanding these mechanisms has critical implications for intervention strategies, suggesting that addressing hearing loss through rehabilitation may potentially modify multiple risk pathways for cognitive decline and dementia.

### 3.7. Is There a Synergistic Effect Between Obstructive Sleep Apnea and Hearing Loss on Cognitive Decline?

Although OSA and hearing loss have largely been examined as independent conditions in the research literature, growing evidence points to shared biological mechanisms that may link them and jointly amplify cognitive risk. Intermittent hypoxemia, systemic low-grade inflammation, oxidative stress, endothelial dysfunction, and microvascular injury are each capable of damaging both the cochlea and cortical regions involved in memory, attention, and executive function. Whether combined exposure to OSA and hearing loss produces additive or synergistic acceleration of cognitive decline remains an open question, however, since direct longitudinal studies designed to test this interaction are still limited in number and scope [32].

The evidence linking OSA to sensorineural hearing loss provides a foundation for understanding their potential interaction. Multiple studies have shown that the incidence of hearing impairment in the OSA group is higher than in control groups, and the average hearing threshold of OSA patients is elevated compared to controls [53,54,55]. The proposed mechanism involves cochlear ischemia resulting from chronic intermittent hypoxemia. The main pathology of hearing loss in patients with OSA is cochlear ischemia due to chronic intermittent hypoxemia. Additionally, sleep apnea may promote hearing impairment through ischemia and inflammation of the cochlea, with a dose–response relationship observed between nadir oxygen saturation and severity of hearing impairment.

The cochlea is particularly vulnerable to hypoxic injury due to its unique anatomical features. The apnea attacks in OSA can lead to reduced oxygen content in cerebrovascular circulation and in the arteries supplying the cochlea. The cochlea and acoustic nerve receive blood supply from terminal arteries without collateral circulation; hence, they are very sensitive to reduced oxygen concentration. The labyrinthine artery, an end artery that supplies the inner ear, is devoid of collaterals and hence is highly vulnerable to ischemic effects. This vulnerability creates a mechanistic link whereby OSA-induced hypoxia may simultaneously damage both auditory and cognitive neural pathways [83].

Studies assessing combined risk factor effects demonstrated dose–response relationships between cumulative risk burden and cognitive outcomes.

The neurobiological plausibility for synergistic effects between OSA and hearing loss is strengthened by overlapping brain regions affected by both conditions. OSA and hearing loss both impact temporal lobe structures, including the hippocampus, which is critical for memory consolidation [84]. Both hearing loss and cognitive decline can independently or synergistically affect the neural processes underlying evoked responses. Hearing loss may primarily impact the earlier sensory components due to degraded auditory input, whereas cognitive decline might more significantly affect later components associated with memory and attention processes. The convergence of damage from both conditions on these vulnerable brain regions may accelerate the trajectory toward dementia [85,86].

Furthermore, both OSA and hearing loss contribute to social isolation and reduced cognitive stimulation, factors known to independently accelerate cognitive decline. Although it is more commonly accepted that vascular and metabolic OSA-associated comorbidities may lead to stroke and vascular dementia, an alternative role of cerebrovascular pathology in Alzheimer’s disease pathogenesis is now recognized, with both pathologies synergistically promoting cognitive decline. The combined social withdrawal resulting from hearing difficulties and OSA-related fatigue may compound these effects [21].

The clinical implications of a potential synergistic relationship are substantial. Fourteen broadly defined modifiable risk factors were significantly associated with dementia, including sensory loss and sleep disturbance. Prevention strategies should consider approaches that reduce the incidence and severity of these risk factors through health promotion, identification, and early management. If OSA and hearing loss interact synergistically, integrated screening programmes targeting both conditions could yield disproportionate benefits for dementia prevention [87].

Despite these theoretical foundations, direct empirical evidence examining the specific interaction between OSA and hearing loss on cognitive outcomes remains limited. OSA is not yet universally recognized as a modifiable risk factor for dementia. Moreover, it remains unclear whether treatment of OSA can slow cognitive decline or reduce the risk of dementia. Future research should employ longitudinal designs that simultaneously assess OSA severity, hearing function, and cognitive trajectories to determine whether the combined presence of both conditions accelerates decline beyond what would be expected from additive effects [19].

While direct evidence for synergistic effects between OSA and hearing loss on cognition is currently limited, the substantial overlap in pathophysiological mechanisms, shared risk factors, and converging effects on vulnerable brain structures provides a compelling rationale for investigating this relationship. The recognition that multiple modifiable risk factors accumulate to increase dementia risk supports integrated clinical approaches that simultaneously address both OSA and hearing loss as potentially synergistic contributors to cognitive decline.

### 3.8. What Is the True Global Burden of Early-Onset Cognitive Decline Attributable to Obstructive Sleep Apnea and Hearing Loss in Low- and Middle-Income Countries?

The global burden of cognitive decline attributable to modifiable risk factors has become an urgent public health concern, particularly in low- and middle-income countries (LMICs) where the majority of affected individuals reside and where healthcare resources remain limited. Both OSA and hearing loss may contribute to this burden, with their impact on cognitive decline being particularly concerning in resource-limited settings.

The prevalence of OSA in LMICs presents a substantial and growing challenge. Studies from rural populations have revealed concerning rates of undiagnosed OSA [4,5]. In the hypertensive and overweight/obese population, the prevalence of OSA is about 29.3%. Given the ongoing obesity epidemic, increases in OSA prevalence are certain globally, and, in particular, in the two most populous nations, China and India [1,10,88].

The hearing loss burden in LMICs is equally substantial and disproportionately affects these regions. Eighty per cent of all hearing loss occurs in low- and middle-income countries. The causes of hearing loss in these settings differ from those in high-income countries. These variations could be attributed to population-level differences in the causative mechanisms of hearing loss in LMICs, such as a higher prevalence of infectious disease-related hearing loss. Furthermore, the majority of persons with hearing loss globally reside in low- and middle-income countries; however, only a small proportion of the world’s rehabilitative resources are allocated to these regions, resulting in a vast unmet need. The majority of individuals with hearing loss worldwide reside in low- and middle-income countries, but there is limited information regarding the characteristics of hearing loss in these regions [1,89].

The population attributable fraction (PAF) of dementia from hearing loss is substantial. The population attributable fraction of dementia from any audiometric hearing loss was 32.0% [85]. Recent studies using objective audiometric measurements have revealed that previous estimates significantly underestimated the contribution of hearing loss. Up to 17% of dementia cases in the United States were attributable to moderate or greater audiometric hearing loss in late-life, and there was evidence for a greater proportion attributable among males relative to females. In LMICs, the PAF from modifiable risk factors, including hearing loss, may be even higher.

Quantifying this regional disparity, recent epidemiological estimates reveal that the weighted PAF for dementia attributable to preventable risk factors stands at 39.5% in China and 41.2% in India, escalating to an alarming 55.8% within Latin American cohorts. These figures underscore that the potential impact of targeted lifestyle and medical interventions is substantially more profound in resource-limited environments than in high-income countries.

Access to diagnostic and treatment services for both conditions remain severely limited in LMICs. Addressing OSA in low- and middle-income countries presents unique challenges due to limited access to diagnostic resources, treatment options, and sleep medicine specialists. For OSA specifically, beyond the issue of diagnosis, there is a paucity of treatment options for OSA in low- and middle-income countries. In addition to the worldwide need to develop novel specific pharmacological interventions against OSA, there is a need to make continuous positive airway pressure therapy, the current gold-standard treatment, available in and feasible for populations in developing countries, especially in rural areas where electricity supply is expensive and unreliable [90].

Similarly, the hearing healthcare infrastructure in LMICs is grossly inadequate. Despite the heavy burden of ear complaints, there is inadequate hearing healthcare delivery in a typical LMIC community. This highlights the need for urgent improvement in hearing healthcare. The challenges extend to access to hearing devices. In Africa, for example, less than 3% of persons who need hearing aids are able to access these devices. Key barriers have been identified, including a lack of trained personnel, the high cost of many existing devices marketed in LMICs and limited public awareness of the benefits of hearing assistive technologies [10,88].

The dementia epidemic in LMICs continues to expand at an alarming rate. Much of the increase will be in developing countries. Already 60% of people with dementia live in LMICs, but by 2050, this will rise to 71%. Furthermore, dementia is substantially underdiagnosed in these regions. In high-income countries, only 20–50% of dementia cases are recognized and documented in primary care. This ‘treatment gap’ is certainly much greater in LMICs, with one study in India suggesting 90% remains undiagnosed [1,85].

The economic consequences of this burden are profound. It is concerning to note that 63–73% of the total costs are incurred in developing countries, and approximately 10% of all these costs are among the people who have hearing loss in the sub-Saharan African region [88]. The potential for prevention in LMICs is substantial. The dementia prevention potential in India, China, and this sample of Latin American countries is large and greater than in high-income countries. In Brazil alone, the ten preventable risk factors for dementia accounted for 50.5% of the Population Attributable Fraction. Hearing loss (14.2%), physical inactivity (11.2%), and hypertension (10.4%) accounted for the highest PAF among all the risk factors [3,91].

Prevention efforts targeting modifiable risk factors, including OSA and hearing loss, offer significant potential [92]. Efforts to prevent or delay dementia onset are likely to have the greatest benefit in LMICs, where most dementia occurs, and where individuals are most likely to encounter poverty, inequality, and limited access to health care.

### 3.9. What Are the Optimal Timing and Target Populations for Intervention to Maximize Neuroprotective Benefits?

The identification of modifiable risk factors for dementia has prompted urgent questions regarding when and in whom interventions should be implemented to achieve maximal neuroprotective benefits. Both OSA and hearing loss represent conditions that develop gradually over time, with pathophysiological consequences that may accumulate long before clinical symptoms of cognitive impairment manifest. Evidence has accrued pointing to dementias as late-life clinical phenotypes that begin as midlife pathologies [93]. Effective prevention, therefore, may need to begin in midlife to succeed. Understanding the optimal timing and target populations for intervention is essential for maximizing the efficiency and effectiveness of dementia prevention strategies [94].

For OSA, evidence suggests that intervention during midlife may be particularly important for preventing cognitive sequelae. In a randomized study of 33 patients with OSA aged 71.3 ± 5.5 years, 3 months of CPAP improved short-term memory, working memory, selective attention, and executive functions [40]. More recently, preliminary evidence showed that CPAP treatment delayed the age of MCI onset by approximately 10 years, whereas age at MCI onset in participants reporting treated OSA was like the non-OSA group. These findings suggest that early treatment of OSA may substantially delay the onset of cognitive impairment [21].

The heterogeneity of cognitive effects of OSA across different age groups underscores the importance of tailored intervention strategies. Broadly, in middle-aged adults, OSA is often associated with mild impairment in attention, memory and executive function. The great disparity in OSA prevalence, the possibility of varying comorbidities, and the distinct phenotypic presentation in young and middle-aged vs. older adults pose an alluring question for sleep and ageing researchers. Understanding the relationship between OSA and risk for AD is crucial to better tailor preventive and treatment strategies [95].

For hearing intervention, the ACHIEVE trial has provided critical insights into which populations benefit most from treatment. These findings suggest that a hearing intervention might reduce cognitive change over 3 years in populations of older adults at increased risk for cognitive decline, but not in populations at decreased risk for cognitive decline. The hearing intervention did not reduce 3-year cognitive decline in the primary analysis of the total cohort. However, a prespecified sensitivity analysis showed that the effect differed between the two study populations that comprised the cohort. This finding has profound implications for targeting hearing interventions [61].

Findings from the ACHIEVE study suggest that older adults at increased risk for cognitive decline and dementia who also have hearing loss may benefit the most from this hearing intervention within three years. In a subgroup of older adults with hearing loss who were at higher risk of cognitive decline, using hearing aids for three years cut cognitive decline in half. These results indicate that individuals with elevated baseline dementia risk, rather than the general population, represent the optimal target for hearing intervention aimed at cognitive protection [67].

On this ground, identifying high-risk populations for targeted intervention has become a research priority. A larger effect was observed in the younger age group (65–70 years) and those with the lowest level of education, who had a higher baseline risk, suggesting that targeting high-risk populations might be more effective. Several risk profiling approaches have been developed to identify individuals most likely to benefit from intervention [96]. The risk profiles were associated with sociodemographic characteristics such as age, education, and region, pointing toward structural differences in dementia risk exposure across the population. These findings lay a foundation for developing more targeted, subgroup-specific prevention strategies.

The timing of OSA treatment relative to the development of Alzheimer’s disease pathology is an important consideration. Because there are low base rates of Aβ in those in midlife, trials aiming to detect changes in AD biomarkers in midlife would need to incorporate very long treatment and follow-up periods or focus on those aged > 60 years, where Aβ deposition is more evident [97]. In summary, treatment of OSA may slow the onset of dementia and AD, yet optimal design of randomized trials presents several challenges. Selection of study population and setting may require identifying individuals who are presymptomatic but at elevated risk of future dementia. The window of opportunity for intervention may exist before substantial amyloid accumulation has occurred [97].

Research supports the value of early intervention for hearing loss across the lifespan. The benefit of early intervention for language development increased as hearing loss increased. Children whose amplification started at age 24 months had poorer language than those whose amplification started at 3 months [98]. While this research pertains to childhood hearing loss, analogous principles may apply to age-related hearing loss in adults. Earlier intervention before prolonged auditory deprivation may preserve neural pathways critical for cognitive function [98].

The concept of cognitive reserve provides a theoretical framework for understanding timing effects. Prevention is about policy and individuals. Contributions to the risk and mitigation of dementia begin early and continue throughout life, so it is never too early or too late. Interventions that enhance cognitive reserve in midlife may provide greater protection against dementia than those initiated after cognitive decline has begun [99].

## 4. Discussion

This review findings underscore that both OSA and hearing impairment represent clinically meaningful and potentially modifiable conditions associated with cognitive deterioration and dementia. It is critical, however, to acknowledge that the robustness of the underlying evidence varies substantially between these two disorders. While large epidemiological cohorts and randomized intervention data supports the clinical and preventive value of addressing hearing loss, the evidence surrounding OSA remains more heterogeneous. This is related to residual confounding, variable diagnostic thresholds, differences in cognitive endpoints, and inconsistent adherence to long-term therapy. The findings of this review underscore that both obstructive sleep apnea (OSA) and hearing impairment represent clinically meaningful, modifiable risk factors closely tied to cognitive deterioration and dementia. It is critical, however, to acknowledge that the robust nature of the underlying data varies substantially between these two disorders. While the clinical and preventive value of addressing hearing loss is backed by solid epidemiological cohorts and high-quality intervention trials, the data surrounding OSA remains considerably more fragmented. This heterogeneity stems primarily from residual confounding factors, shifting diagnostic boundaries across clinics, and the clinical challenge of inconsistent patient adherence to long-term therapies. 

The global burden of these conditions is substantial and disproportionately affects low- and middle-income countries, where diagnostic and treatment resources remain severely limited. Addressing this disparity represents both a public health imperative and an opportunity to prevent millions of dementia cases worldwide.

Several methodological limitations of the present review have to be noted. First, the reliance on predominantly observational data inherently restricts our ability to establish firm causal relationships. Second, a high degree of heterogeneity was observed across the literature, particularly regarding baseline cohort demographics, specific diagnostic criteria for both OSA and auditory deficits, follow-up intervals, and the statistical adjustments applied for potential confounders. Third, because cognitive decline shares an overlapping risk profile with both sleep disorders and hearing loss including advanced age, metabolic syndromes, and vascular comorbidities the impact of residual confounding cannot be entirely ruled out.

Furthermore, our search strategy excluded non-English literature, which may introduce a geographic or language bias. Lastly, given the narrative nature of this study, a formal meta-analysis and standardized risk-of-bias grading were not performed.

In conclusion, while both OSA and hearing impairment are highly prevalent issues closely linked to cognitive deterioration and dementia, the current quality of evidence varies considerably depending on the specific outcomes and therapeutic interventions evaluated. The role of hearing loss as a modifiable risk factor for dementia is supported by robust data, though biologically compelling demands more rigorous longitudinal research and structured intervention trials.

To clarify whether early clinical management can successfully alter long-term cognitive decline, subsequent research must focus on joint OSA-hearing loss phenotypes, rely on standardized cognitive assessments, utilize objective exposure tracking, and pinpoint the optimal therapeutic window for both CPAP compliance and auditory rehabilitation.

## Figures and Tables

**Table 1:** Summary of the main evidence linking obstructive sleep apnea, hearing loss, and early-onset cognitive decline.

Topic	Main Type of Evidence	Main Findings	Strength of Evidence	Main Limitations	Interpretation
OSA and cognitive decline	Observational cohort studies and meta-analyses	OSA and sleep-disordered breathing have been associated with an increased risk of cognitive impairment, mild cognitive impairment (MCI), dementia, and earlier onset of neurocognitive decline.	Moderate	Residual confounding; heterogeneous OSA definitions; variable cognitive endpoints; inconsistent adjustment for comorbidities.	The association is supported by several studies, but causality remains uncertain.
OSA severity and cognitive outcomes	Cross-sectional and cohort studies using AHI and hypoxemia-related metrics	AHI shows inconsistent associations with cognition, whereas hypoxemia-related measures may better reflect neurocognitive risk.	Low to moderate	AHI does not capture event duration, cumulative hypoxic burden, sleep-stage specificity, or arousal intensity.	Hypoxic burden may be more informative than AHI alone, but further validation is needed.
Mechanisms linking OSA to cognitive decline	Experimental, neuroimaging, and translational studies	Intermittent hypoxia, sleep fragmentation, endothelial dysfunction, neuroinflammation, oxidative stress, and impaired glymphatic clearance may contribute to brain injury.	Moderate mechanistic plausibility	Mechanistic evidence does not prove clinical causality; pathways are overlapping and difficult to isolate.	Biologically plausible pathways support, but do not prove, causal inference.
CPAP and cognition	Randomized trials, observational studies, and neuroimaging studies	CPAP may improve attention and vigilance, particularly with good adherence and early treatment; effects on memory and established cognitive impairment are less consistent.	Moderate for selected cognitive domains; low for dementia prevention	Short follow-up; poor adherence; heterogeneous populations and cognitive endpoints.	CPAP may provide cognitive benefit in selected patients, but evidence for reversing established impairment remains limited.
Hearing loss and cognitive decline	Longitudinal cohort studies and meta-analyses	Hearing loss is consistently associated with faster cognitive decline and increased dementia risk.	Moderate to high for association	Potential residual confounding; reverse causality; heterogeneity in hearing assessment.	The association is robust, but direct causality remains debated.
Mechanisms linking hearing loss to neurodegeneration	Epidemiological, neuroimaging, and mechanistic studies	Cognitive load, social isolation, reduced stimulation, cortical reorganization, and shared pathology may explain the association between hearing loss and cognitive decline.	Moderate mechanistic plausibility	Mechanisms may coexist; the relative contribution of each pathway remains uncertain.	Multiple plausible mechanisms exist, but their causal hierarchy is unresolved.
Hearing aids and cochlear implants	Observational studies, randomized trials, and intervention studies	Hearing rehabilitation may slow cognitive decline, especially in individuals at increased baseline risk; cochlear implants may improve selected cognitive domains.	Moderate	Adherence; timing of intervention; selection bias; limited long-term randomized data.	Early and consistent hearing rehabilitation may be beneficial, particularly in high-risk populations.
Combined OSA and hearing loss	Limited observational and hypothesis-generating studies	Coexistence of OSA and hearing loss may be associated with worse cognitive outcomes than either condition alone.	Low	Few dedicated studies; limited longitudinal evidence; uncertain interaction effects.	A possible additive or synergistic effect is biologically plausible but not yet proven.
Global burden and LMICs	WHO/GBD reports and epidemiological estimates	OSA and hearing loss are highly prevalent and often underdiagnosed or undertreated, especially in low- and middle-income countries (LMICs).	Moderate	Sparse direct data on early-onset cognitive decline attributable to these conditions in LMICs.	Public health relevance is high, but region-specific attributable burden remains uncertain.

## Data Availability

No new data were created or analyzed in this study. Data sharing is not applicable to this article.

## Abbreviations

The following abbreviations are used in this manuscript:

AD	Alzheimer’s Disease
ADNI	Alzheimer’s Disease Neuroimaging Initiative
AHI	Apnea–Hypopnea Index
BMI	Body Mass Index
CPAP	Continuous Positive Airway Pressure
CSF	Cerebrospinal Fluid
EEG	Electroencephalography
GBD	Global Burden of Disease
HR	Hazard Ratio
LMICs	Low- and Middle-Income Countries
MCI	Mild Cognitive Impairment
MoCA	Montreal Cognitive Assessment
OSA	Obstructive Sleep Apnea
PAF	Population-Attributable Fraction
PSG	Polysomnography
REM	Rapid Eye Movement
SANRA	Scale for the Assessment of Narrative Review Articles
WHO	World Health Organization
